# 2-Iodo-4′-Methoxychalcone Attenuates Methylglyoxal-Induced Neurotoxicity by Activation of GLP-1 Receptor and Enhancement of Neurotrophic Signal, Antioxidant Defense and Glyoxalase Pathway

**DOI:** 10.3390/molecules24122249

**Published:** 2019-06-16

**Authors:** Yu-Ting Tseng, Yi-Hong Tsai, Ferenc Fülöp, Fang-Rong Chang, Yi-Ching Lo

**Affiliations:** 1 Department of Pharmacology, School of Medicine, College of Medicine, Kaohsiung Medical University, Kaohsiung 80708, Taiwan; mmship1112@yahoo.com.tw; 2 Graduate Institute of Natural Products, College of Pharmacy, Kaohsiung Medical University, Kaohsiung 80708, Taiwan; lyph0719@hotmail.com (Y.-H.T.); aaronfrc@kmu.edu.tw (F.-R.C.); 3 Institute of Pharmaceutical Chemistry, University of Szeged, Eötvös u. 6, H-6720 Szeged, Hungary; fulop@pharm.u-szeged.hu; 4 MTA-SZTE Stereochemistry Research Group, Hungarian Academy of Sciences, Eötvös u. 6, H-6720 Szeged, Hungary; 5 National Research Institute of Chinese Medicine, Ministry of Health and Welfare, Taipei 11221, Taiwan; 6 Department of Medical Research, Kaohsiung Medical University Hospital, Kaohsiung 80708, Taiwan; 7 Graduate Institute of Medicine, College of Medicine, Kaohsiung Medical University, Kaohsiung 80708, Taiwan

**Keywords:** halogen-containing chalcones, methylglyoxal, neurotrophic effect, antioxidant defense, glyoxalase pathway, neuroprotection

## Abstract

Methylglyoxal (MG) acts as a reactive precursor of advanced glycation end products (AGEs). This compound is often connected with pathologies such as diabetes, neurodegenerative processes and diseases of aging. 2-iodo-4′-methoxychalcone (CHA79), a synthetic halogen-containing chalcone derivative, has been reported its anti-diabetic activity. This study aims to investigate the potential protective capability of CHA79 against MG-mediated neurotoxicity in SH-SY5Y cells. Results indicated CHA79 increased viability of cells and attenuated the rate of apoptosis in MG-exposed SH-SY5Y. CHA79 up-regulated expression of anti-apoptotic protein (Bcl-2) and down-regulated apoptotic proteins (Bax, cytochrome *c*, caspase-3, caspase-9). Moreover, CHA79 significantly up-regulated expression of neurotrophic factors, including glucagon-like peptide-1 receptor (GLP-1R), brain derived neurotrophic factor (BDNF), p75NTR, p-TrkB, p-Akt, p-GK-3β and p-CREB. CHA79 attenuated MG-induced ROS production and enhanced the antioxidant defense including nuclear factor erythroid 2-related factor 2 (Nrf2), HO-1, SOD and GSH. Furthermore, CHA79 attenuated MG-induced reduction of glyoxalase-1 (GLO-1), a vital enzyme on removing AGE precursors. In conclusion, CHA79 is the first novel synthetic chalcone possessing the GLP-1R and GLO-1 activating properties. CHA 79 also exhibits neuroprotective effects against MG toxicity by enhancing neurotrophic signal, antioxidant defense and anti-apoptosis pathway.

## 1. Introduction

Methylglyoxal (MG) is a potent glycating agent that accumulates in chronic hyperglycemic state such as diabetes mellitus (DM) [1]. This compound also has gained research attention due to its ability to induce neurotoxicity [2,3,4]. MG can impair cellular redox homeostasis by inhibiting antioxidant defense and glyoxalases, as well as inducing reactive oxygen species (ROS) [5,6]. Therefore, improving antioxidant defense such as nuclear factor erythroid 2-related factor 2 (Nrf2) mediated signaling and up-regulating glyoxalase system are deeply involved in detoxifying MG [7,8,9].

In addition to antioxidant defense and glyoxalase system, there are still several pathways that have been suggested for their potential on defense against MG toxicity. For example, the enhancing glucagon-like peptide-1 receptor (GLP-1R) pathway, a pharmacological management of DM, has been reported to attenuate MG-caused apoptosis in SH-SY5Y cells [10]. Moreover, lower levels of brain derived neurotrophic factor (BDNF) and tyrosine kinase B receptor (TrkB) are found in hippocampal neurons of MG-accumulated rats [11]. As well as protein kinase B (Akt) and cAMP response element binding (CREB), their involvement in attenuating MG-related learning and memory impairment of aging rats have also been studied [12]. Recently, MG is considered to have a probable linkage to Parkinson’s disease (PD) by making dopaminergic neurons more vulnerable due to its structural similarity and chemical reactivity with dopamine oxidation products [1,4,13]. Therefore, a pharmacological method of detoxifying MG may assist in the development of novel drugs targeted at improving PD or other neurodegenerative diseases (NDs).

Chalcones (1,3-diaryl-2-propen-1-ones) are a class of aromatic enones classified into the flavonoid family. They are open-chain flavonoid compounds that possess a basic skeleton of A, B rings linked with α, β-unsaturated carbonyl system [14,15]. Different plant-derived types of chalcones have been isolated containing a variety of substituted elements, including methyl, methoxy and hydroxy substituents on both of the aromatic rings [16]. The most advantageous and attractive factor in producing chalcones and their derivatives are that they can be reacted via a one-step protocol using an aldol condensation between a benzaldehyde and an acetophenone in the presence of a base. Derivatives can be easily and directly recrystallized to reach purity, with yield usually up to 80% [17].

**Figure 1:**
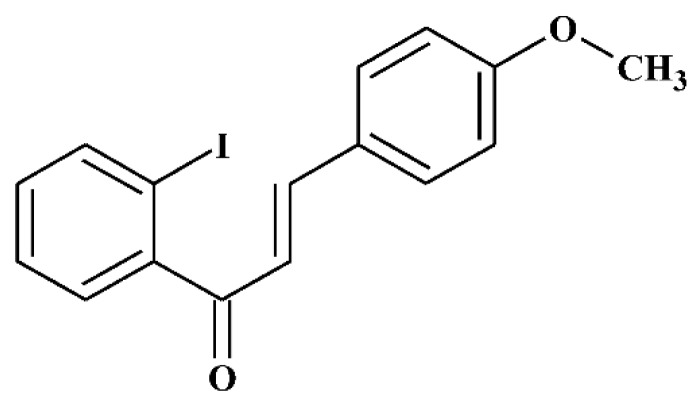
Chemical structure of 2-iodo-4′-methoxychalcone (CHA79).

According to the study on chalcone’s structure-activity relationship (SAR) in our previous study, the presence of a methoxy group on ring B and the halogen substituents on ring A on chalcone seem to be most effect in DM-related disorders. In particular, a chalcone derivative with iodo substitution at position 2 on A-ring, which never occurred in natural resources and named 2-iodo-4′-methoxychalcone (CHA79, Figure 1), has been shown to be more potent than other anti-diabetic drugs. It is noteworthy that halogen-containing chalcone derivatives are very rare in the plant kingdom, but can easily be scaled up synthetically as mentioned above [15,16]. CHA79 is suggested to act as a 5′-adenosine-monophosphate-activated protein kinase (AMPK) activator and possesses anti-diabetic activity in the literature [16]. However, whether CHA79 potentially possesses benefits in MG-induced neurotoxicity has not yet been studied. This paper is the first to reveal the dopaminergic neuronal protection of CHA79 against MG toxicity in SH-SY5Y cells and investigate the underlying mechanisms.

## 2. Results

### 2.1. CHA79 Mitigates Neuronal Apoptosis Accompany with Modulation of Anti-Apoptotic and Apoptotic Signals in MG-Treated SH-SY5Y Dopaminergic Neurons

**Figure 2:**
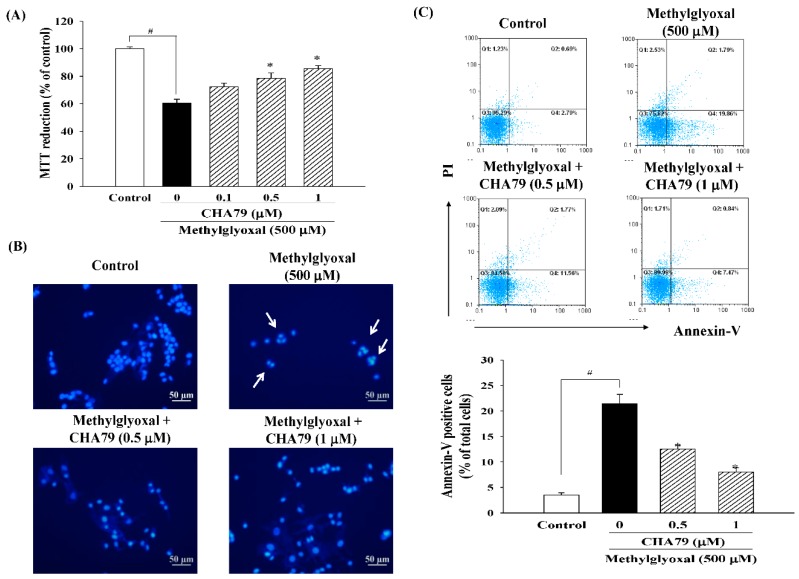
Effects of CHA79 on cell viability (**A**), nuclear condensation (**B**), and Annexin V-positive cell numbers (**C**) in MG-treated SH-SY5Y dopaminergic neurons. Cells were pre-treated with CHA79 (0.1–1 μM) for 1 h, and MG (500 μM) was then treated for 24 h. Cell viability was determined by MTT assay. Nuclear condensation (white arrow) was determined by Hoechst 33342 and observed by a fluorescent microscope. Scale bar = 50 μM. Annexin V-positive cell numbers were counted by flow cytometer and were represented as the percentage of total cell numbers. # *p* < 0.05 versus the control group (vehicle control: 0.1% DMSO). * *p* < 0.05 versus the MG group.

**Figure 3:**
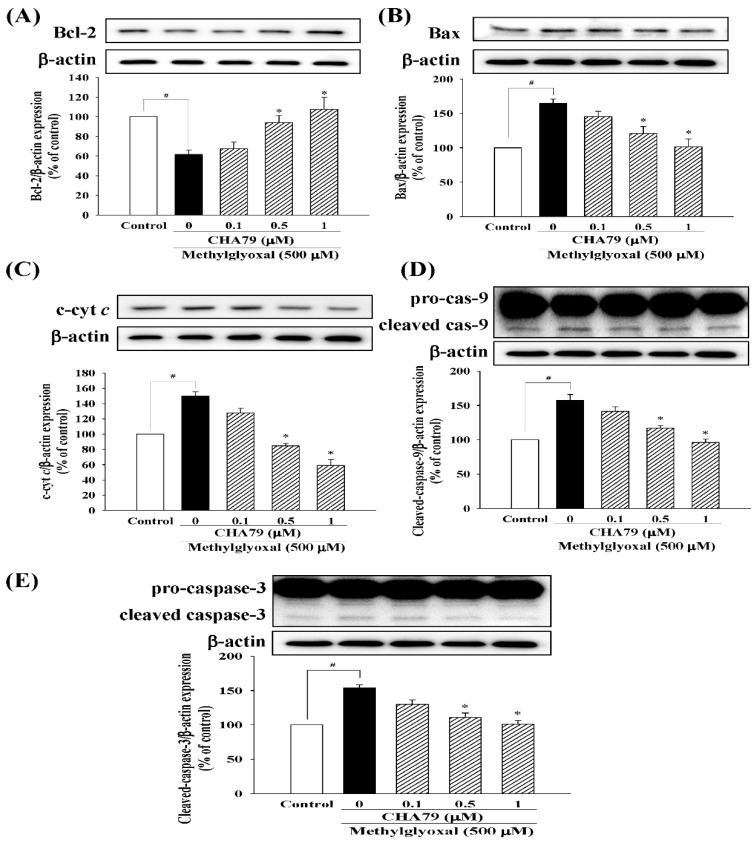
Effects of CHA79 on expressions of Bcl-2 (**A**), Bax (**B**), cytosolic cytochrome *c* (c-cyt *c*) (**C**), cleaved caspase-9 (**D**), and cleaved caspase-3 (**E**) in MG-treated SH-SY5Y dopaminergic neurons. Cells were pre-treated with CHA79 (0.1–1 μM) for 1 h, and MG (500 μM) was then treated for 24 h. Densitometry analyses are presented as the relative ratio of protein/β-actin protein, and are represented as percentages of control group (vehicle control: 0.1% DMSO). Bars represent the mean ± SEM (*n* = 6). # *p* < 0.05 versus the control group (vehicle control: 0.1% DMSO). * *p* < 0.05 versus the MG group.

We first confirmed that CHA79 did not cause cytotoxicity under concentration of 0.1 to 1 μM in SH-SY5Y cells (as shown in the supplemental materials, Figure S1), and we performed the study within this dose range. MTT (3-(4,5-dimethylthiazol-2-yl)-2,5-diphenyl-tetrazolium bromide) assay was used to examine whether CHA79 possesses protection against MG caused neurotoxicity. Result from MTT data demonstrated that MG decreased cell viability to 60.61 ± 2.70% of control group, while pretreatment of 0.5 and 1 μM of CHA79 improved cell viability to 78.59 ± 3.68 and 85.50 ± 2.22% of the control group in under MG exposure (Figure 2A). The anti-apoptotic effects of CHA79 on MG-treated SH-SY5Y dopaminergic neurons were also examined by using Hoechst 33342 and Annexin-V staining, respectively. The Hoechst 33342 staining result showed that CHA79 (0.5 and 1 μM) attenuated MG-induced nuclear condensation (indicated by white arrows) in SH-SY5Y cells (Figure 2B). Pretreatment of 0.5 and 1 μM of CHA79 attenuated numbers of Annexin V-positive cell from 21.46 ± 1.78% (MG group) to 12.49 ± 0.82 and 8.05 ± 0.80% respectively (Figure 2C). Similar results were also found in related proteins evaluation. Results indicated that MG decreased B-cell lymphoma 2 (Bcl-2), which is known as anti-apoptotic protein; however, CHA79 (0.5 and 1 μM) improved Bcl-2 protein level in MG-treated SH-SY5Y cells (Figure 3A). CHA79 (0.5 and 1 μM) also attenuated MG-induced pro-apoptotic BCL2 associated X (Bax) (Figure 3B) protein level in SH-SY5Y cells. MG-induced cytosolic cytochrome *c* increase was also down-regulated by CHA79 (Figure 3C). While MG induced cleavage of caspase-9 and caspase-3, western blot results showed CHA79 (0.5 and 1 μM) could significantly attenuate protein expressions of cleaved caspase-9 (Figure 3D) and cleaved caspase-3 (Figure 3E) in MG-treated SH-SY5Y cells.

### 2.2. CHA79 Enhances GLP-1R, BDNF, and Related Neurotrophic Signals in MG-Treated SH-SY5Y Dopaminergic Neurons

**Figure 4:**
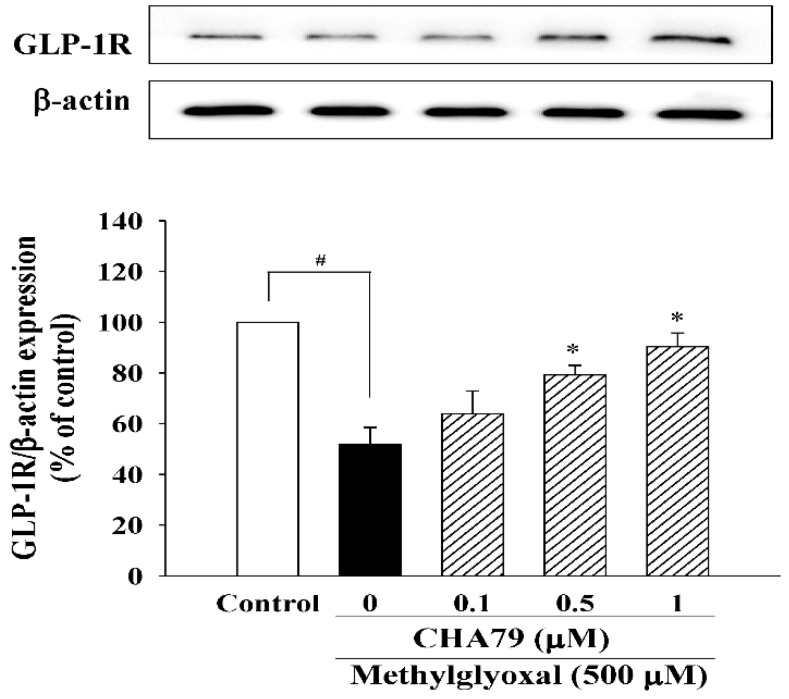
CHA79 up-regulated the expression of GLP-1R in MG-treated SH-SY5Y dopaminergic neurons. One-hour pre-treatment with CHA79 (0.1–1 μM) followed by 24 h MG (500 μM) exposure was carried out. WB data are presented as % of control follow by normalization with internal standard (β-actin). Data are shown as mean ± SEM (*n* = 6). # *p* < 0.05 versus the control group (vehicle control: 0.1% DMSO). * *p* < 0.05 versus the MG group.

**Figure 5:**
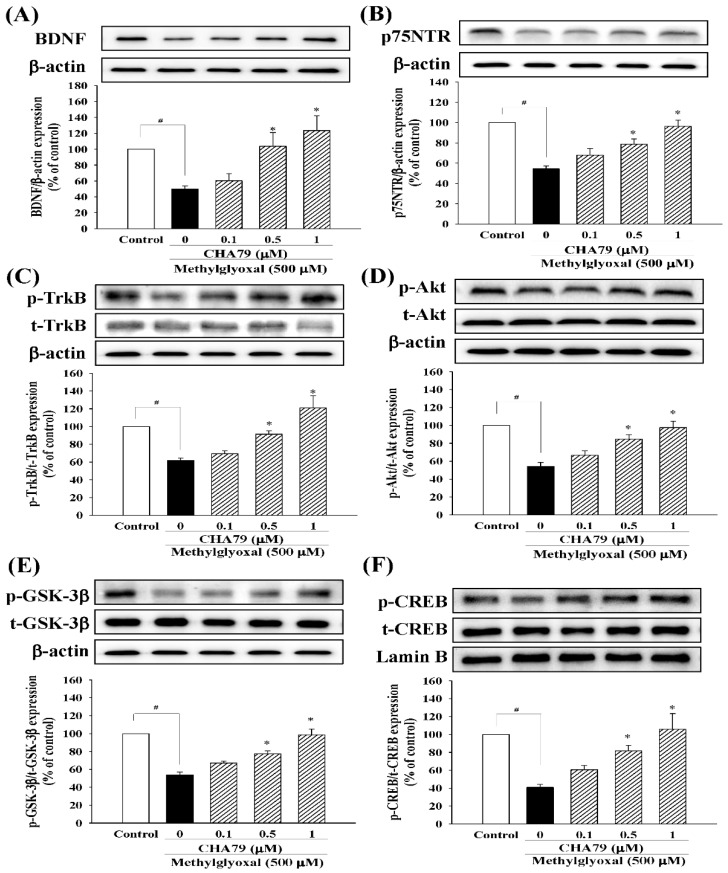
CHA79 up-regulated BDNF (**A**), p75NTR (**B**), p-TrkB (**C**), p-Akt (**D**), p-GSK-3β (**E**), and p-CREM (**F**) expression in MG-treated SH-SY5Y dopaminergic neurons. One-hour pre-treatment with CHA79 (0.1–1 μM) followed by 24 h MG (500 μM) exposure was carried out. WB data are presented as % of control follow by normalization with internal standard (β-actin or Lamin B). Data are shown as mean ± SEM (*n* = 6). # *p* < 0.05 versus the control group (vehicle control: 0.1% DMSO). * *p* < 0.05 versus the MG group.

We investigated whether CHA79 has an effect on an important anti-diabetic target with neuroprotective potential, GLP-1R. The results indicated that CHA79 (0.5 and 1 μM) attenuated the down-regulation of GLP-1R caused by MG in SH-SY5Y cells (Figure 4). Investigations into BDNF were also carried out, as reduced BDNF has been postulated as an important cause on dopaminergic neurons loss in PD by causing a lack of trophic support. Our tests showed CHA79 could up-regulate BDNF and related neurotrophic pathways in MG-treated SH-SY5Y dopaminergic neurons. Reduced BDNF level was found in MG-treated SH-SY5Y cells, whileCHA79 (0.5 and 1 μM) led to an improvement of BDNF level (Figure 5A). The expressions of two common neurotrophin receptors that bind BDNF, p75NTR (Figure 5B) and p-TrkB (Figure 5C), were also down-regulated in MG-treated SH-SY5Y cells. CHA79 (0.5 and 1 μM) showed up-regulation on p75NTR (Figure 5B) and p-TrkB (Figure 5C) expressions in MG-treated SH-SY5Y cells. Moreover, in SH-SY5Y cells, CHA79 (0.5 and 1 μM) attenuated MG-induced down-regulation on phosphorylation of Akt (Figure 5D), glycogen synthase kinase-3 beta (GSK-3β) (Figure 5E) and CREB (Figure 5F).

### 2.3. CHA79 Relieves Oxidative Stress via Regulation of Antioxidant Defense and Glyoxalase Pathway in MG-Treated SH-SY5Y Dopaminergic Neurons

**Figure 6:**
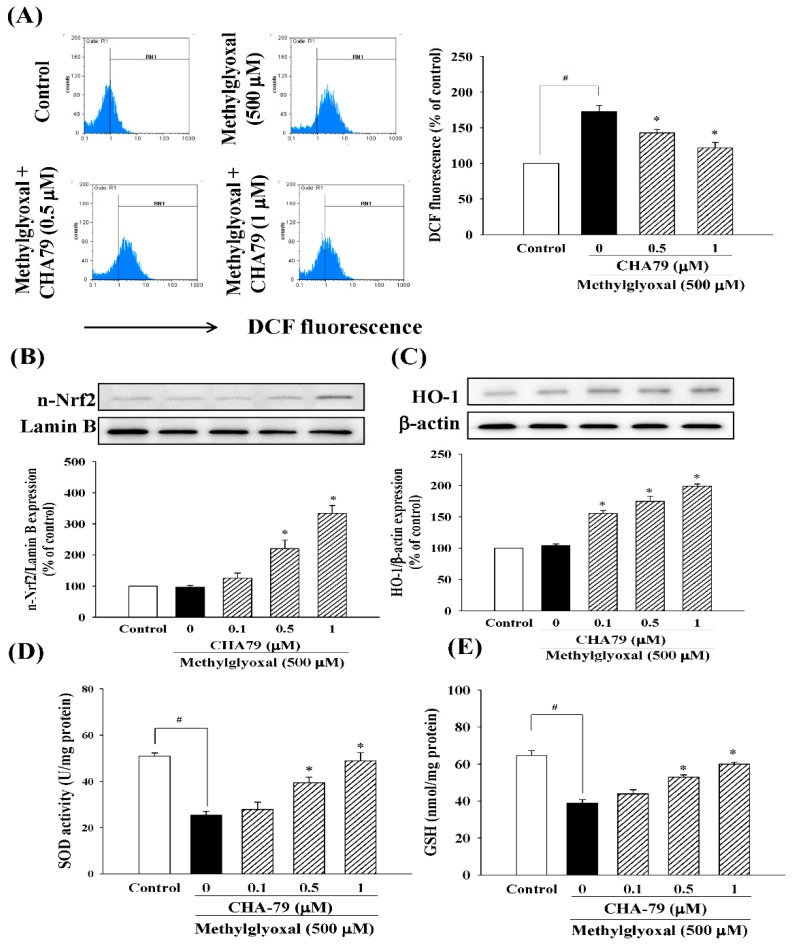
CHA79 down-regulated ROS production (**A**), up-regulated nuclear-Nrf2 (n-Nrf2) expression (**B**), HO-1 expression (**C**), SOD activity (**D**), total GSH level (**E**) in MG-treated SH-SY5Y dopaminergic neurons. One-hour pre-treatment with CHA79 (0.1–1 μM) followed by 24 h MG (500 μM) exposure was carried out. Fluorescent DCF was measured a by flow cytometer as the indicator of ROS production. WB data are presented as % of control follow by normalization with internal standard (β-actin or Lamin B). Commercial kits were used for detecting GSH and SOD. Data are shown as mean ± SEM (*n* = 6). # *p* < 0.05 versus the control group (vehicle control: 0.1% DMSO). * *p* < 0.05 versus the MG group.

**Figure 7:**
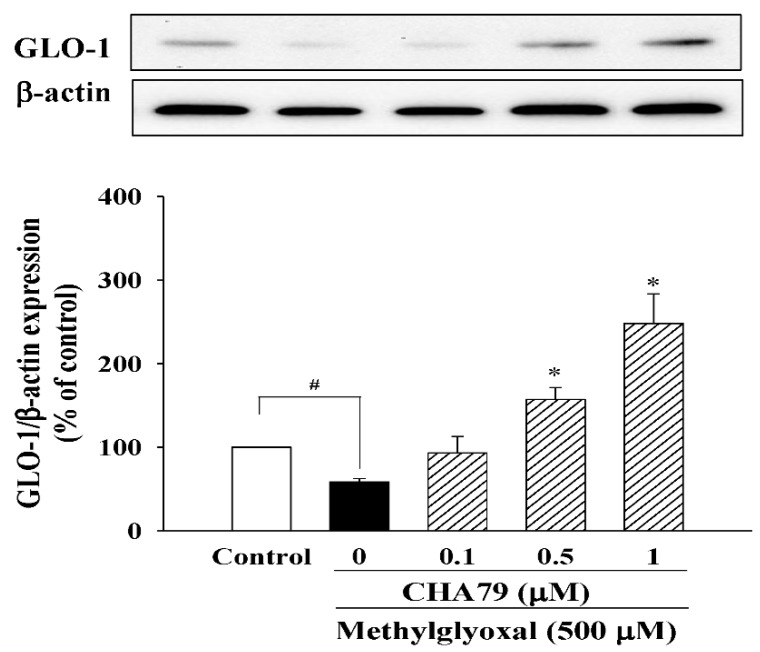
CHA79 up-regulated expression of GLO-1 in MG-treated SH-SY5Y dopaminergic neurons. One-hour pre-treatment with CHA79 (0.1–1 μM) followed by 24 h MG (500 μM) exposure was carried out. WB data are presented as % of control follow by normalization with internal standard (β-actin). Data are shown as mean ± SEM (*n* = 6). # *p* < 0.05 versus the control group (vehicle control: 0.1% DMSO). * *p* < 0.05 versus the MG group.

Next, we evaluated the anti-oxidative ability of CHA79 in MG-treated SH-SY5Y dopaminergic neurons. As shown in our results, MG significantly increased ROS production to 172.60 ± 8.80% of control group in SH-SY5Y cells, while pretreatment of CHA79 (0.5 and 1 μM) could attenuate MG-induced ROS overproduction to 142.80 ± 5.08 and 121.54 ± 7.75% of control group respectively (Figure 6A). The important anti-oxidant defense Nrf2/heme oxygenase-1 (HO-1) pathway, superoxide dismutase (SOD) activity, and total glutathione (GSH) level were also examined in the present study. Results indicated that CHA79 up-regulated both nuclear Nrf2 (n-Nrf2) expression (Figure 6B) and HO-1 expression (Figure 6C) in MG-treated SH-SY5Y cells, confirming the improvement of Nrf2/HO-1 pathway by CHA79. Additionally, there was decreased SOD activity by MG treatment from 50.93 ± 1.38 to 25.34 ± 1.69 U/mg protein, while 0.5 and 1 μM of CHA79 pretreatment improved SOD activity to 39.38 ± 2.49 and 48.95 ± 3.55 U/mg protein in MG-treated SH-SY5Y cells (Figure 6D). Similarly, total GSH level was attenuated by MG from 64.57 ± 2.71 to 38.84 ± 1.89 nmol/mg protein in SH-SY5Y cells, however, CHA79 (0.5 and 1 μM) leveled it up to 52.90 ± 1.25 and 59.96 ± 1.16 nmol/mg protein respectively (Figure 6E). Moreover, we also examined the effects of CHA79 on glyoxalase pathway, an important mechanism for MG detoxification with glyoxalase-1 (GLO-1) as the key enzyme in the defense system. In MG-treated SH-SY5Y cells, a reduced expression of GLO-1 was found. Pretreatment of CHA79 (0.5 and 1 μM) showed significant improvement on GLO-1 protein level in MG-treated neurons (Figure 7).

## 3. Discussion

DM is a common metabolic disorder with multi-system clinical and pathological manifestations. MG accumulation is a well-recognized pathologic feature of DM, and studies have reported its importance in various DM complications and age-related diseases [18,19,20,21]. MG is known to be a potent compound derived primarily from the metabolism of glucose. As glucose is the main energy source of brain, neuronal cells in brain are more exposed to higher levels of MG than other cell types. MG has been suggested to have a role in NDs including PD and Alzheimer’s diseases (AD). MG shares similar structure and reactivity with dopamine oxidation products [1,4,13] and has been reported to interact with PD-associated α-synuclein [22]. These findings support that MG exposure is probably an important risk factor of PD. In addition, MG has been reported to exacerbate the neurotoxicity of AD-associated β-amyloid (Aβ) by glycation in primary hippocampal neurons and Tg2576 mice [23]. Accordingly, we set out to explore compounds targeting MG detoxication in this study. The present study demonstrated a novel therapeutic approach of CHA79 on neuroprotection against MG in SH-SY5Y dopaminergic neurons. We also revealed the capabilities of CHA79 on enhancing GLP-1R, neurotrophic signal, antioxidant defense and glyoxalase pathway under MG exposure.

CHA79 is a chalcone derivative able to be synthesized using a one-step protocol with high purity and yield in our previous study [15]. In this prior study, chalcones with hydroxy, chloro, bromo, and iodo substitutions substitution at position 2 of the A-ring show better antidiabetic activity. Their improvement on glucose consumption is superior to pioglitazone and rosiglitazone, two clinically used antidiabetic drugs [15]. CHA79 (with iodo substitution) is also reported to lower glucose level in adipocytes (3T3-L1 cells), myotubes (differentiated C2C12 cells) and high-fat-diet-treated animals [15,16].

Recent literature reveals a GLP-1R agonist, liraglutide, attenuates hippocampal neuronal death in intracerebroventricular streptozotocin (STZ)-injected rats; the effect is accompanied with hyperphosphorylation of AMPK [24]. We understand that CHA79 is capable of inducing AMPK activation [16]; however, in our knowledge, this study is the first to investigate and demonstrate the ability of CHA79 to enhance GLP-1R in MG-treated SH-SY5Y cells. This study also appears to be the first to discover GLP-1R activating effects of chalcones.

Increasing studies have characterized the neuroprotective role of GLP-1R in cellular and animal models of PD using pharmacological management [25,26]. Activation of GLP-1R also has been reviewed as a novel treatment strategy for PD recently for its neuroprotective and neurotrophic roles [27]. From this background of literature, we hypothesize that CHA79 should be able to promote neurotrophic support in MG-treated SH-SY5Y dopaminergic neurons.

BDNF is a neurotrophic major factor in synaptic plasticity, neuronal differentiation and neuronal survival. This neurotrophin is also an important molecular target for PD treatment [28]. The present results revealed that CHA79 could up-regulate protein levels of BDNF as well as p75NTR and p-TrkB, two receptors with high affinity to BDNF, in neuronal SH-SY5Y exposed to MG. p75NTR is a receptor for all mature neurotrophins and immature proneurotropins, and therefore, plays diverse roles in regulating neuronal survival and degeneration [29,30]. However, when co-expressed with TrkB, p75NTR tends to potentiate the survival pathway. p75NTR can also enhance TrkB autophosphorylation in response to its preferred ligands such as BDNF [31]. Binding of BDNF to TrkB induces receptor autophosphorylation and activates TrkB signaling pathway. The p-TrkB provides phosphorylation-dependent recruitment sites for initiation of downstream signaling that mediates neuronal survival and outgrowth [32]. Such as Akt, GSK-3β and CREB are all characterized phosphorylation-dependent downstream signaling contributes to neuronal survival or neurite outgrowth in prior studies [26,33,34,35]. As shown in our results, CHA79 up-regulated expressions of phosphorylated form Akt, GSK-3β, as well as CREB in neuronal SH-SY5Y under MG exposure. These results suggest the capability of CHA79 on enhancing BDNF and BDNF-activated neurotrophic signal.

In additional to the neurotrophic effects of CHA79, its anti-oxidative ability has also been determined in the present study. As we know, chalcones are a type of open-chain flavonoids; compounds that belong to the group of polyphenols. Polyphenols are well-known for their effective anti-oxidative ability. Therefore, flavonoids receive great attention in this regard for decades, and their antioxidant capability has been reviewed [36,37]. Our results demonstrated this chalcone derivative, CHA79, also exhibited anti-oxidative property via down-regulating ROS production under MG exposure in SH-SY5Y cells. Nrf2-activated pathway is a widely recognized antioxidant and cytoprotective regulation. Nrf2 can be activated and translocated to nucleus whereas redox state is changed. Activation of Nrf2 is capable of restoring the redox homeostasis by improving antioxidant or other cytoprotective enzymes [38]. HO-1 is one of the important antioxidant enzymes regulated transcriptionally via Nrf2. Many compounds possess Nrf2/HO-1 improvement is reported to be effective against inflammation and oxidative stress-associated diseases [39,40,41]. Moreover, enzymatic SOD and non-enzymatic GSH can also be produced by Nrf2 transcription. They are reported to be responsible for protecting neuronal cells from oxidative stress [39,42,43,44]. In this study, we were able to demonstrate that CHA79 is capable of up-regulating n-Nrf2 and HO-1 expressions, SOD activity and total GSH level in MG-exposed SH-SY5Y.

Recently, the effects of flavonoids on MG detoxification through modulation of glyoxalase pathway have gained some research interest [37,45]. The glyoxalase pathway is an important pathway that contributes to MG detoxification with GLO-1 as the key enzyme in regulating the rate-limiting step of MG metabolism [46]. With the existence of cofactor GSH, GLO-1 is able to attenuate AGEs formation by promoting MG clearance. The important role of GLO-1 in MG clearance are also been suggested in diabetic encephalopathy and nephropathy models of animal [47,48]. Therefore, GLO-1-based therapeutic approach is also suggested to be a valuable target for MG-related aging and diseases [9]. Our results showed improvement of CHA79 on protein expression of GLO-1 in MG-treated SH-SY5Y cells. As discussed above, CHA79 could up-regulate the GSH level. Moreover, the intracellular pool of GSH is also reported as an important co-factor for GLO-1 in glyoxalase pathway [46]. Accordingly, our results revealed the antioxidative capability of CHA79 accompany with regulation on Nrf2 associated antioxidant defense and glyoxalase pathway.

As MG-induced loss of neurotrophic support and increase of oxidative stress can eventually lead to neuronal apoptosis, our results further confirmed CHA79 mediated anti-apoptotic activity against MG toxicity in this study. MG is reported to induce apoptosis accompany with down-regulation of Bcl-2 and up-regulation of Bax in hippocampal neurons [2]. This effect is known to influence mitochondrial outer membrane permeabilization, allowing cytochrome *c* redistributes from mitochondria to cytosol. The outcome is that downstream effectors such as caspase-9 and caspase-3 are further cleaved and activated [49]. Our results showed that CHA79 attenuated expressions of Bax, cytosolic cytochrome *c*, cleaved caspase-9, and cleaved caspase-3 in MG-treated SH-SY5Y cells; whereas Bcl-2 was increased by CHA79 treatment. MG leads an increase in apoptotic cell numbers but was able to be attenuated by CHA79 treatment. According to our results, CHA79 could protect dopaminergic SH-SY5Y against apoptotic damage caused by MG.

In conclusion, we identified CHA79 as a GLP-1R-activating compound, effective in attenuating MG neurotoxicity with neurotrophic, antioxidant and glyoxalase pathway-improving capabilities. This study is the first to reveal the capacity of chalcone derivative on GLP-1R improvement. Most importantly, the simple synthetic method and high purity and yield of CHA79 support its feasibility for commercial production. This study opens perspectives for using CHA79 as a novel drug candidate in suppressing MG toxicity in the context of PD or other NDs.

## 4. Materials and Methods

### 4.1. Chemicals and Reagents

2-iodo-4′-methoxychalcone (CHA79) was synthesized as previously described [15]. Chemical reagents, such as MG, hoechst 33342, 2′,7′-dichloro-dihydrofluorescein diacetate (H_2_DCF-DA), 3-(4,5-dimethylthiazol-2-yl)-2,5-diphenyl-tetrazolium bromide (MTT), etc., were obtained from Sigma-Aldrich (St. Louis, MO, USA). Cell culture used materials were from Thermo Fisher Scientific (Waltham, MA, USA). Annexin V-FITC assay kit was from BD Bioscience (San Jose, CA, USA). SOD activity and GSH quantitation kit were from Enzo Life Sciences (Farmingdale, NY, USA). SDS-PAGE used materials were from Bio-Rad (Hercules, CA, USA). All primary and secondary antibodies used in Western blots (WB) are listed in supplemental material (Table S1).

### 4.2. Cell Cultures and Procedure of Drugs Management

ATCC (Rockville, MD, USA) is the source of the present used culture cells (SH-SY5Y human neuroblastoma), and these cells were used as models of dopaminergic neuronal cells after differentiation. Incubation condition of cells is 37 °C in 5% CO_2_. Culture medium used for SH-SY5Y cells was FBS (10%)-containing DMEM (also contains 4 mM glutamine, 100 U/mL penicillin, 100 μg/mL streptomycin, and 0.25 μg/mL amphotericin B). Experiments were carried out within cell passes five to ten. For further experiments, SH-SY5Y cells were differentiated by 6 days incubation with serum-free defined DMEM medium (containing 4 mM glutamine, 100 U/mL penicillin, 100 μg/mL streptomycin, and 0.25 μg/mL amphotericin B) [35]. Dopaminergic toxicity was induced by MG at 500 μM according to our supplemental data (Figure S2) and previous studies [50,51]. One-hour pre-treatment with 0.1% DMSO (vehicle) or 0.1–1 μM CHA79 followed by 24 h MG (500 μM) exposure was carried out.

### 4.3. Evaluation of Cell Viability and Neuronal Apoptosis

MTT assay was carried out for evaluating cell viability. MTT contains a tetrazolium ring, which can be cleaved by dehydrogenases of living cells. Upon cleavage, it forms formazan crystals, which can be solved in organic solvent such as DMSO, and can be detected by microplate reader (Thermo Scientific, Waltham, MA, USA) at absorbance of 560 nm. Briefly, culture medium was removed after indicated drug treatment. Cells were then incubated with 0.5 mg/mL MTT for 3 h in 37 °C. The formazan crystals were dissolved with 100 μL DMSO, and absorbance was read (Thermo Scientific, Waltham, MA, USA). Apoptosis was assessed by fluorescence staining with Hoechst 33342 and AnnexinV-FITC. While DNA condensation/nuclear fragmentation as indicator of cell apoptosis, Hoechst 33342 staining is able to assess apoptosis under fluorescence microscopy (Nikon, Melville, NY, USA). In AnnexinV-FITC experiment, neurons were collected, resuspended in binding buffer and incubated with AnnexinV-FITC for staining. Coulter CyFlow Cytometer (Partec, Canterbury, Kent, UK) was then used to count Annexin V-positive cells as indicator of apoptosis.

### 4.4. Evaluation of Oxidative Stress

ROS production was determined as an indicator of oxidative stress. H_2_DCF-DA staining was used, and ROS production was determined by analyzing DCF (fluorescent product, Enzo Life Sciences Inc., Farmingdale, NY, USA) by Coulter CyFlow Cytometer. After incubation with H_2_DCF-DA (10 μM), neurons were collected. DCF fluorescence was detected within a hundred thousand cells using Cytometer (excitation: 495 nm, emission: 520 nm).

### 4.5. Evaluation of Antioxidant Defense

SOD activity kit and GSH quantitation kit (Enzo Life Sciences Inc., Farmingdale, NY, USA) were used for evaluating antioxidant capability, according to manufacturer’s instructions. Briefly, to determine SOD activity, Master mix and xanthine solution were added to each well that containing protein extract or SOD buffer (control). SOD activity was determined by detecting WST-1 formazan (OD 450 nm; every one-min for ten times), which is conversed from WST-1 with xanthine oxidase. The activity was expressed as units per milligram of protein (U/mg protein). Total GSH level was quantitated by measuring the production of 5-thio-2-nitrobenzoic acid (yellow color) (OD 405 nm; every one-min for ten times) from GSH and DTNB. Briefly, samples were homogenized in 0.5 M perchloric acid and centrifuged at 12,000× *g* for 5 min. The supernatant was collected and neutralized (with 0.1 M phosphate buffer, pH 7.0, and 1 mM EDTA) for detection. Data were calculated by means of a calibration curve and normalized to the protein concentration.

### 4.6. Western Blots (WB)

WB was used for detecting protein expressions. Protein extraction reagent (Cat. No. 78510, Thermo Fisher Scientific) supplemented with protease inhibitor cocktail (Cat. No. 04693132001, Roche) and nuclear protein extraction kit (Cat. No. 78833, Thermo Fisher Scientific) were used for isolating cytosolic and nuclear protein extracts. Proteins (20 μg) were separated on gels (SDS polyacrylamide) and then transferred to membranes (PVDF). TBST that contains non-fat milk (5%) was then used for blocking non-specific binding sites of proteins. Specific primary antibodies that shown in chemical and reagent section and appropriate secondary antibodies were then used for membrane incubation. Quantification on levels of protein was calculated by Image J (NIH, Bethesda, MD, USA).

### 4.7. Statistical Analysis

Mean ± SEM was used for data presentation. All Statistical analyses were performed with InStat version 3.0 (GraphPad Software, San Diego, CA, USA). All pair comparisons were analyzed by ANOVA followed by Dunnett’s test. Differences with *p* < 0.05 were considered statistically significant.

## Supplementary Materials

The following are available online. Figure S1: Effect of CHA79 on cell viability in SH-SY5Y dopaminergic neurons. Figure S2: Effect of MG on cell viability in SH-SY5Y dopaminergic neurons. Table S1: Antibodies used in Western blots.
