# Supplementary figures and images for: 2-Iodo-4′-Methoxychalcone Attenuates Methylglyoxal-Induced Neurotoxicity by Activation of GLP-1 Receptor and Enhancement of Neurotrophic Signal, Antioxidant Defense and Glyoxalase Pathway
Source: Molecules. 2019 Jun 16;24(12):2249. doi: 10.3390/molecules24122249 (PMC6631972; doi:10.3390/molecules24122249)

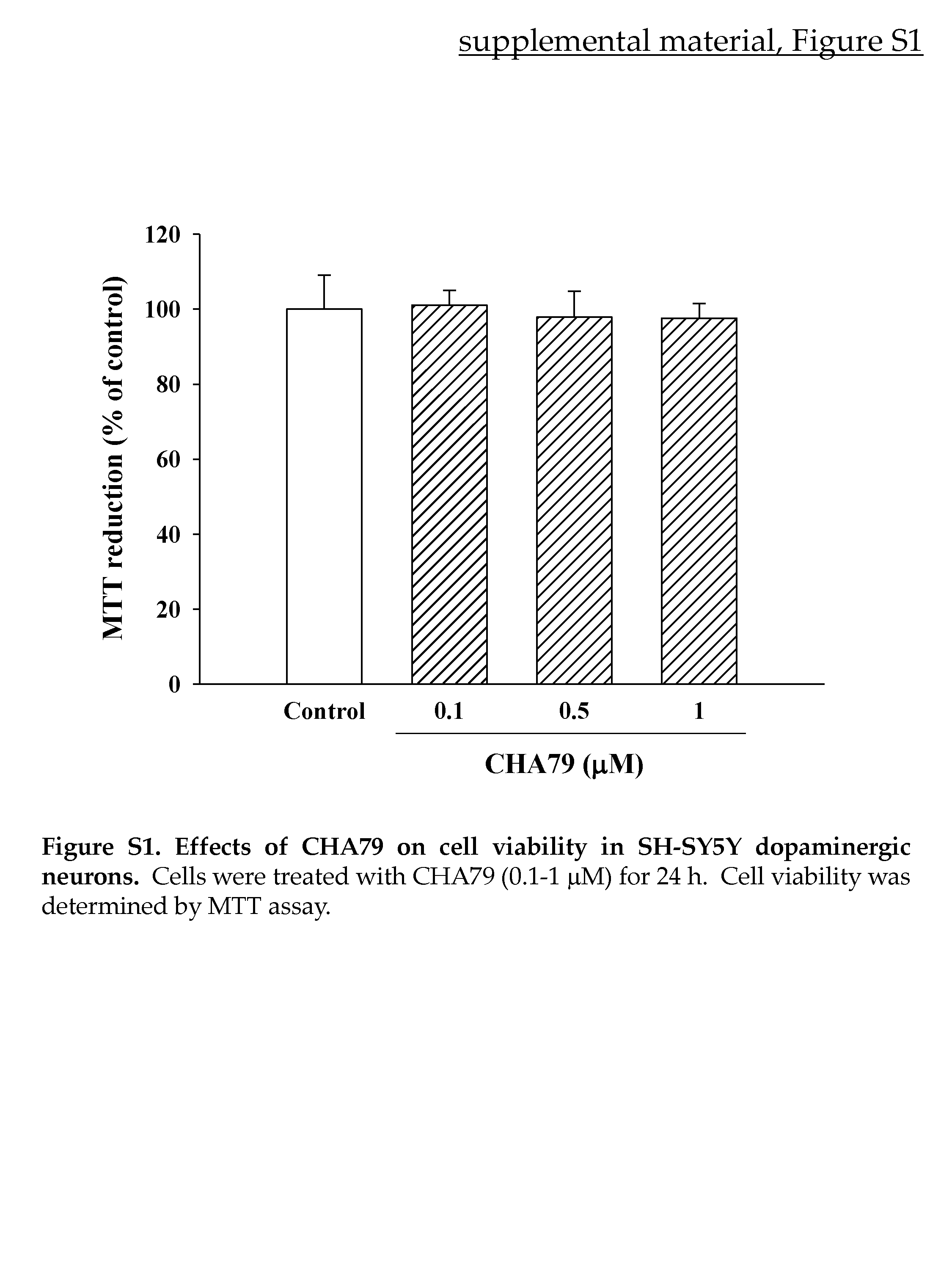

Supplement: Supplementary file 1 [file molecules-24-02249-s001.zip › Fig S1.tiff]

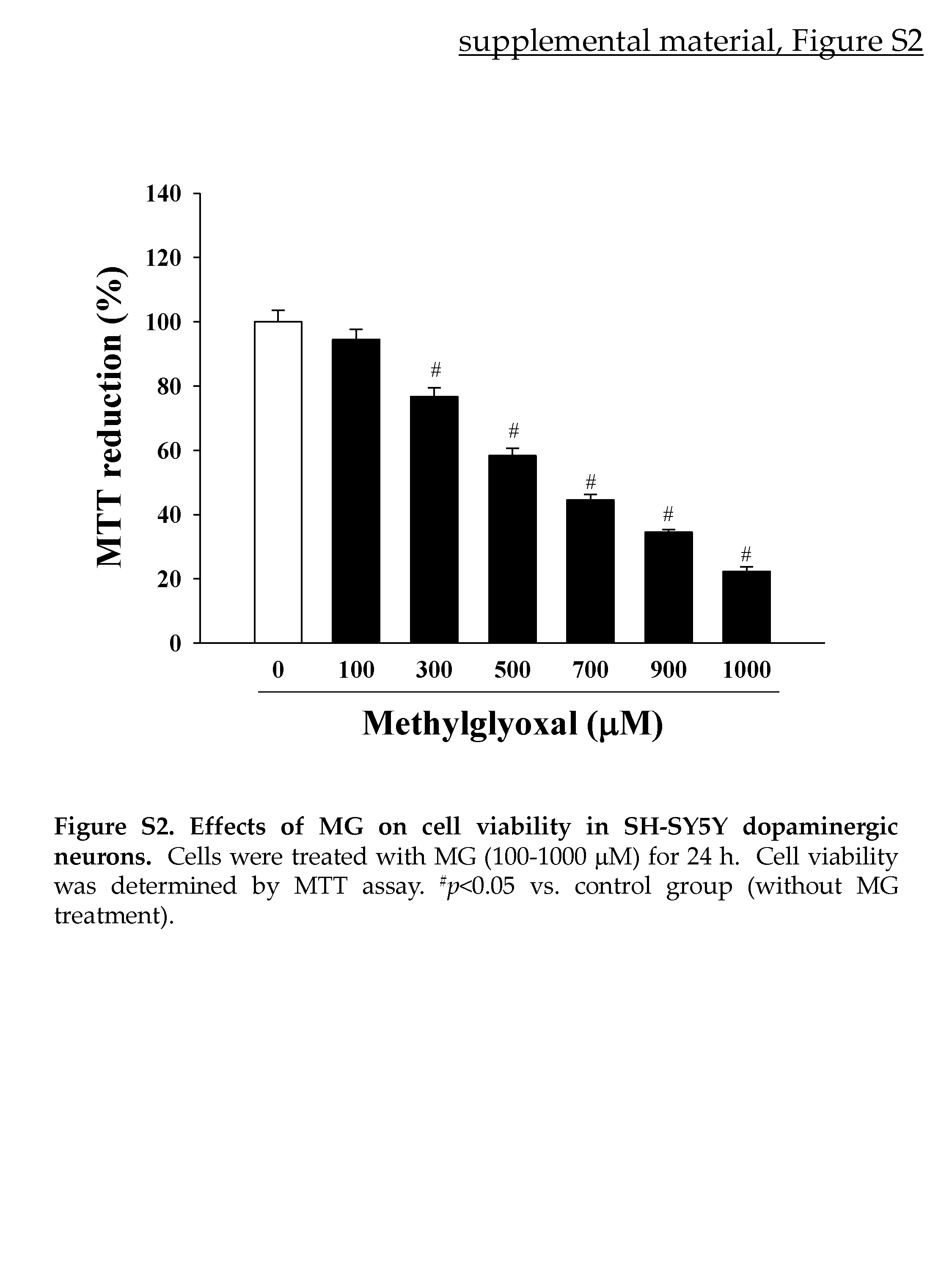

Supplement: Supplementary file 1 [file molecules-24-02249-s001.zip › Fig S2.tiff]

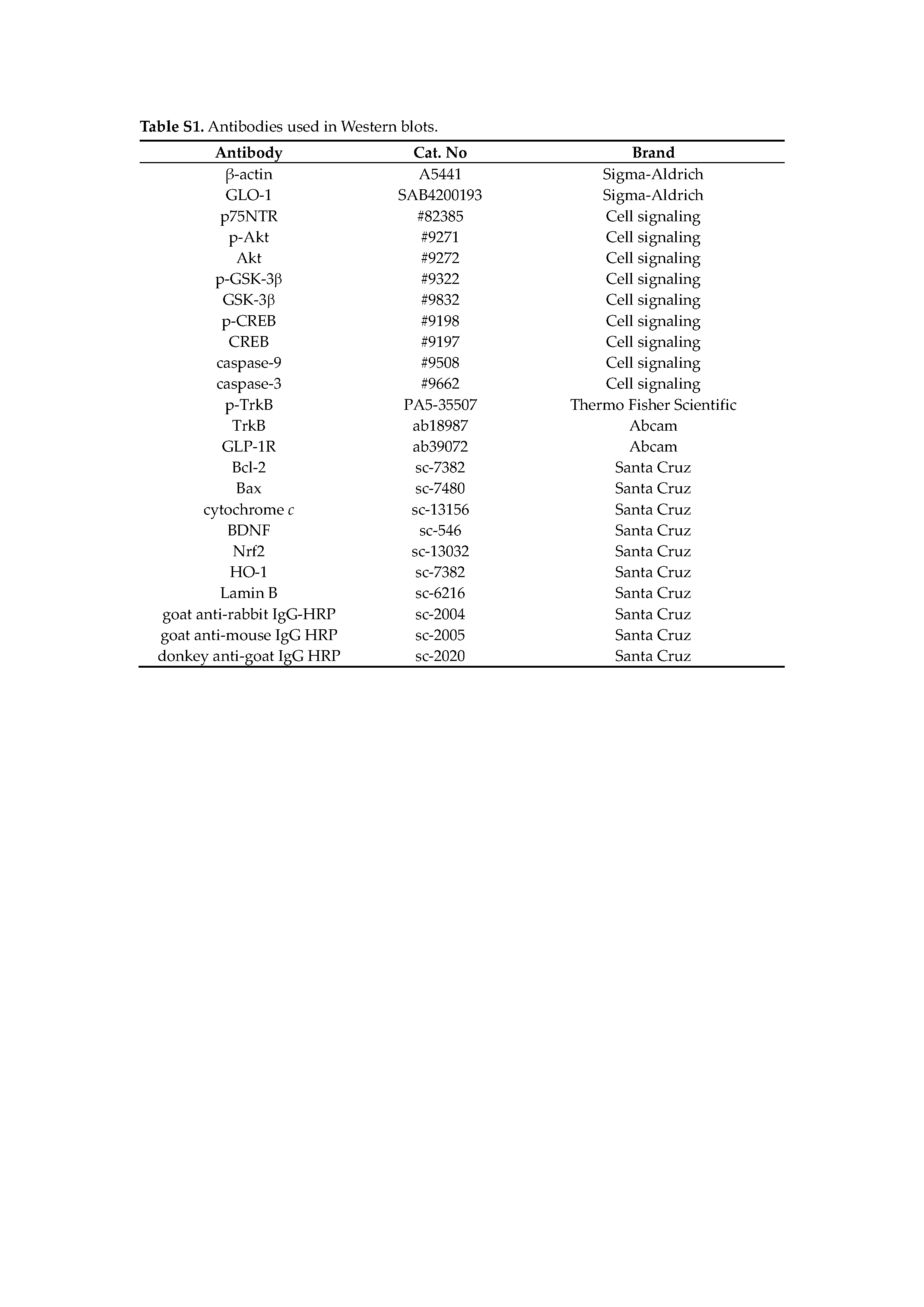

Supplement: Supplementary file 1 [file molecules-24-02249-s001.zip › Table S1.tiff]
